# Mr. Custance, on Yeast

**Published:** 1801-02

**Authors:** Geo. Custance

**Affiliations:** Kidderminster


### *To the Editors of the Medical and Phyſical Journal.*

GENTLEMEN,

THE impartiality with which your Journal is conducted, leaves me no room to doubt of your favouring me with an early inſertion of the following Remarks, in reply to Mr. Groſe, on the ſubject of "Yeaſt in Typhus."

Mr. Groſe is much to be commended for inveſtigating the ſubject by ſuch experiments as would enable him to draw any uſeful and ſatisfactory inferences from them; but I think all he has done and ſaid would have met the public eye with equal propriety, if he had not indirectly charged me with being a "Perſecutor,"* and inſinuated a ſuſpicion of my candour and veracity, by obſerving, that all who have written in your Journal, on the ſubject of yeaſt in typhus, "with the exception of *one* gentleman, have *impartially confessed* the beneficial effects." A conduct like this is unworthy a man, who profeſſes to be ſearching after truth, and is equally indifferent whether it be ultimately found by himſelf or his neighbour, provided he do but get into poſſeſſion of it. The paſſage in my former letter, which ſeems to have given offence to Mr. Groſe, is that wherein I mention the purgative effects produced by the yeaſt in the caſes which I related.† Authors ſeldom have juſtice done them, when ſelect paſſages alone are conſidered by their Readers; it is only by a comprehenſive view of the *whole* that the true ſenſe of any publication is to be obtained. I remember when a literary character *was tried a few years ago by one of our Univerſities*, *for publiſhing an offensive book*, he complained that the Proſecutor, by ſelecting paſſages, deſtroyed the ſenſe of it, and made him ſpeak things he never intended. "In this way," ſaid he, "I can prove that the Scriptures command you to commit ſuicide; for in one part of the Bible it is ſaid, 'Ahitophel went and hanged himſelf;' and in another, 'Go thou, and do likewiſe." Thus has Mr. Groſe certainly miſconceived the purport of my former letter, by fixing his eye too long upon a particular paſſage; whether owing to the obſcurity of my ſtyle, or his own inattention, is not for me to determine. But I flatter myſelf, if Mr. Groſe will oblige me ſo far as to give my letter another peruſal, he will plainly diſcover that my chief deſign is to obviate the *danger of empiricism* in general, and not to *persecute*, with unrelenting malignity, the judicious exhibition

* See Mr. Groſe's Letter in No. XXII.

† See Poſtſcript to Mr. Groſe's Letter in No. XXI.

exhibition of yeaft; for I even allowed, "that the fixed air in balm may occafionally have fome good effect in putrid difeafes." My objections were, and ftill are, againft the *indiscriminate* ufe of yeaft *alone* in putrid fevers, and holding it out amongft the vulgar, in the moft unqualified manner, as an infallible fpecific; as was done in the Armenian and Monthly Magazine, referred to in my letter. Is it poffible that Mr. Grofe himfelf can believe that a perfon "in the laft ftage of putrid fever," was feen the next morning after taking fome yeaft, "walking in his garden," apparently well? I do not doubt the veracity of the author of this affertion, but I call his judgment in queftion. If fuch were the effects of the yeaft, I cannot believe that the patient was in the laft ftage of putrid fever, efpecially when it is confidered, that he was "an old man upwards of 70."*

Now, as the Armenian or Methodifts Magazine is very extenfively circulated amongft the lower orders in all parts of the kingdom, furely there is a propriety in condemning the *unqualified* encomiums of any medicine as a remedy for putrid fevers. It is obfervable, however, that even in *these strong cases related by the* Rev. Mr. Cartwright, *bark* was conjoined with the ufe of yeaft, and therefore the conclufion that the latter was a fpecific, is not juft; the cure may as well be attributed to the bark. So likewife in the folitary cafe with which Mr. Grofe has favoured the public in No. XXI. the patient "was fponged all over with cold vinegar, and directed the free ufe of yeaft and—*wine.*" So far then as we may judge from what Mr. Grofe has told us, even he has not trufted to his favourite remedy. I doubt not, many of your readers will attribute the fuccefs of this cafe as much to the vinegar and wine as to the yeaft. The ftatement of the cafe, as given by Mr. G. appears to me very vague and unfatisfactory. He tells us, that the boy was attacked on the "10th of July, with the ufual fymptoms of typhus." On the third day, that is, on the 12th, the fponging and ufe of yeaft appear to have been begun. From the 13th "he became evidently better." "In two days after the yeaft was difcontinued."—"On the confideration of the wonderful change which fo rapidly fucceeded the adminiftration of the yeaft," fays Mr. G. "I cannot but imagine it to be the beft remedy in putrid fevers ever yet difcovered." Are we to conclude from this relation, that Mr. G's patient was cured of a putrid fever within five days from its firft attack? If I can underftand Mr. G. right, this is the conclufion he wifhes to be drawn.

* See the Armenian Magazine for Auguft 1799.

drawn. A pretty extenſive practice amongſt perſons generally afflicted with this diſorder, has never afforded me an opportunity of ſeeing a typhus fever terminate in this ſhort ſpace of time. I have ſeldom ſeen petechiæ appear earlier than the 5th, 6th, or 7th day. The criſis does not often take place earlier than the 14th or 17th day; and in the caſes which prove fatal, I have generally obſerved the fever to run on till the 21ſt day. Not that any general rule is invariably obſerved; but I have never ſeen a true typhus, with ſymptoms of putridity, finiſh its courſe in *five* days.

Mr. G. wiſhes to explain the action of the yeaſt on the living ſyſtem, from the known properties of fixed air in ſweetening putrid meat; and aſks, whether ptyaliſm is not excited as well by the external application of mercury, as by the internal uſe of it? Doubtleſs, it is; but in both caſes the effect is produced by the mercury being received into the circulating ſyſtem; and therefore the application of mercury to the ſkin, for the purpoſe of abſorption, is not analogous to the chemical combination of fixed air with putrid meat; conſequently, his reaſoning is inconcluſive.

Having now endeavoured to juſtify the ſentiments I have before obtruded on the public, I take my leave of the ſubject, having no deſign of making your Journal (which will, I am ſure, be employed in a more profitable manner) the vehicle of controverſy. I am, &c.

*Kidderminster, Dec.* 6, 1800. GEO. CUSTANCE.

A contagious fever, of the moſt malignant kind, appeared in London and Weſtminſter, during the months of November and December laſt. A delirium often commenced as ſoon as the third day; and a petechial eruption, though more florid than uſual, appeared on the breaſt and trunk. The other ſymptoms differed little from thoſe uſually obſerved in jail or contagious fevers.—A female patient, afflicted with this formidable diſeaſe, and with permanent delirium, was admitted into the Weſtminſter Hoſpital during the laſt month, and, as uſual, put on the generous and cordial plan; but in a few days neither food nor medicine could be adminiſtered, on account of the repugnance of the patient. In this deſperate ſituation I directed the apothecary to procure ſome freſh yeaſt, and to dilute it if neceſſary, of which a table ſpoonful was to be given every four hours. The patient took it without difficulty; it agreed with the ſtomach and bowels, and in three days an evident amendment was obſerved. The patient is now perfectly cured of the fever, and no other remedy, not even wine, was employed in conjunction with the yeaſt. T. B.

